# Improved Multiplex Ligation-Dependent Probe Amplification Analysis Identifies a Deleterious *PMS2* Allele Generated by Recombination with Crossover Between *PMS2* and *PMS2CL*

**DOI:** 10.1002/gcc.21966

**Published:** 2012-05-14

**Authors:** Annekatrin Wernstedt, Emanuele Valtorta, Franco Armelao, Roberto Togni, Salvatore Girlando, Michael Baudis, Karl Heinimann, Ludwine Messiaen, Noemie Staehli, Johannes Zschocke, Giancarlo Marra, Katharina Wimmer

**Affiliations:** 1 Division of Human Genetics, Medical University InnsbruckAustria; 2 Institute of Molecular Cancer Research, Faculty of Medicine, University of ZurichSwitzerland; 3 Department of Gastroenterology, Ospedale Santa Chiara, Azienda Provinciale per I Servizi SanitariTrento, Italy; 4 Department of Pathology, Ospedale Santa Chiara, Azienda Provinciale per I Servizi SanitariTrento, Italy; 5 Institute of Molecular Life Sciences, Faculty of Science, University of ZurichSwitzerland; 6 Department of Biomedicine, Research Group Human Genetics, University of BaselSwitzerland; 7 Department of Genetics, Medical Genomics Laboratory, University of Alabama at BirminghamBirmingham, AL

## Abstract

Heterozygous *PMS2* germline mutations are associated with Lynch syndrome. Up to one third of these mutations are genomic deletions. Their detection is complicated by a pseudogene (*PMS2CL*), which – owing to extensive interparalog sequence exchange – closely resembles *PMS2* downstream of exon 12. A recently redesigned multiplex ligation-dependent probe amplification (MLPA) assay identifies *PMS2* copy number alterations with improved reliability when used with reference DNAs containing equal numbers of *PMS2*- and *PMS2CL*-specific sequences. We selected eight such reference samples – all publicly available – and used them with this assay to study 13 patients with PMS2-defective colorectal tumors. Three presented deleterious alterations: an *Alu*-mediated exon deletion; a 125-kb deletion encompassing *PMS2* and four additional genes (two with tumor-suppressing functions); and a novel *deleterious* hybrid *PMS2* allele produced by recombination with crossover between *PMS2* and *PMS2CL,* with the breakpoint in intron 10 (the most 5′ breakpoint of its kind reported thus far). We discuss mechanisms that might generate this allele in different chromosomal configurations (and their diagnostic implications) and describe an allele-specific PCR assay that facilitates its detection. Our data indicate that the redesigned *PMS2* MLPA assay is a valid first-line option. In our series, it identified roughly a quarter of all *PMS2* mutations. © 2012 Wiley Periodicals, Inc.

## INTRODUCTION

Lynch syndrome (MIM#120435) is the most common heritable cause of neoplastic disease in the large intestine. Formerly referred to as hereditary nonpolyposis colorectal cancer, this syndrome accounts for approximately 2-3% of all colorectal malignancies and is associated with an increased risk for cancer at other sites as well (Cunningham et al., 2001; Lynch and de la Chapelle, 2003; Hampel et al., 2008; Lynch et al., 2009). It is caused mainly by heterozygous germline mutations in the mismatch repair (MMR) genes, in most cases *MSH2* (∼40% of cases) or *MLH1* (∼50%) (Liu et al., 1996; Peltomaki, 2003)*.* A third MMR gene, *MSH6*, has been found mutated in ∼10% of cases (Berends et al., 2002; Hendriks et al., 2004; Plaschke et al., 2004). Another essential MMR gene, *PMS2*, was previously thought to play only a minor role in colorectal cancer predisposition. However, in a large series of unselected colorectal carcinomas, evidence of primary alteration of *PMS2* (i.e., immunohistochemical findings of isolated loss of PMS2 protein expression with normal expression of its heterodimeric partner MLH1 and of the other MMR proteins) was approximately as common as loss of MSH2 (Truninger et al., 2005). In this study and others (Clendenning et al., 2008; Senter et al., 2008), heterozygous *PMS2* mutations displayed lower penetrance than mutations involving other MMR genes, as reflected by later onset of colorectal cancer (average: 59 years vs. 45 years in *MLH1*/*MSH2* mutation carriers) and weaker family histories of Lynch-syndrome associated cancers. Consequently, patients with these mutations were unlikely to meet the Amsterdam II criteria (Vasen et al., 1999).

Mutational analysis of *PMS2*, which is located on chromosome 7 (band p22), is complicated by the existence of 15 *PMS2* pseudogenes on the same chromosome. One of these, a transcribed pseudogene known as *PMS2CL*, lies on 7p22 itself, just ∼0.7 Mb centromeric to the functional gene. *PMS2CL* is the result of the inverted duplication of a 100-kb repeat element that includes the 3′ region of *PMS2* containing exons 9–15*.* The pseudogene contains six of these *PMS2* exons (9 and 11-15) but lacks exon 10 owing to an *Alu-*mediated 2.7-kb deletion (De Vos et al., 2004).

Recombination involving these two duplicons has led to considerable sequence homogenization at their centers and to a lesser extent at their extremities (Hayward et al., 2007). Recombination with crossover and/or gene-conversion between the paralogs situated at the inner ends of the duplicons produces hybrid *PMS2* alleles that contain *PMS2CL*-derived sequences (as defined by NCBI RefSeq NC_000007.13) as well as hybrid *PMS2CL* alleles with sequences derived from *PMS2* (according to RefSeq NM_000535.5). The transfer involves mainly sequences lying between intron 12 and the 3′ ends of the two paralogs – in terms of coding sequences, those of exons 13–15 (Hayward et al., 2007; Ganster et al., 2010; van der Klift et al., 2010). The *PMS2CL* sequences of these exons are almost always synonymous variants (with respect to those of the functional gene). The only exception is a missense alteration (p.N775S) in *PMS2CL* exon 14. However, hybrid *PMS2* alleles containing this variant represent 4–25% of all *PMS2* alleles found in the general population, depending on the ethnic group (Ganster et al., 2010). Therefore, the vast majority of hybrid *PMS2* alleles identified thus far are classified as nondeleterious.

Because of the very high prevalence of hybrid *PMS2* and *PMS2CL* alleles (van der Klift et al., 2010), the 3′ regions of the gene and pseudogene cannot be reliably distinguished on the basis of sequence differences with respect to their respective NCBI RefSeqs. This is a serious limitation for genomic DNA (gDNA)-based assays used for *PMS2* mutation analyses [e.g., exon sequencing to detect point mutations and multiplex ligation-dependent probe amplification (MLPA) for the identification of deletions/amplifications].

The shortcomings of gDNA-based sequencing of *PMS2* exons 11-15 can be effectively circumvented with recently developed RNA-based strategies, such as complementary DNA (cDNA) sequencing (Etzler et al., 2008; van der Klift et al., 2010), or with long-range PCR (Vaughn et al., 2010). New solutions are also needed for the detection of copy number changes, above all deletions, which appear to account for up to 33% of all *PMS2* mutations (van der Klift et al., 2010; Vaughn et al., 2010). The MLPA assay kit used until recently for this purpose (the P008-A1 *PMS2* MLPA kit, MRC Holland, Amsterdam, The Netherlands) contained only *PMS2*-specific probes for exons 13-15, and this led to both false-negative and false-positive results for these exons due to the presence of hybrid alleles in many gDNA samples. To address these problems, the *PMS2* MLPA kit has recently been redesigned. The new version (kit P008-B1) contains gene- and pseudogene-specific probes for exons 11-15, as well as nonspecific probes for exons 12-15, which hybridize to gene and pseudogene sequences and thus detect a total of four DNA copies in the genome. These modifications have substantially improved the reliability of the assay, specifically from exons 12/13 downwards (Vaughn et al., 2011). Owing, however, to wide interindividual variability in the distribution of *PMS2* and *PMS2CL* sequences downstream of exon 12, copy number variations (CNVs) can be accurately detected only when each run includes reference DNA samples known to contain two gene-specific copies and two pseudogene-specific copies of exons 11-15 sequences.

This study illustrates the pivotal role played by these reference DNA samples and describes eight publicly available samples that can be used with the new *PMS2* MLPA kit. We used this approach to identify the presence of *PMS2* deletions in germline DNA from 13 patients whose colorectal tumors were suspected to be caused by *PMS2*-related Lynch syndrome. In three cases, MLPA yielded evidence of deleterious *PMS2* alterations, which were subsequently verified and further characterized with other methods. Two were deletions (one of which encompassed the entire *PMS2* gene and at least two other genes with tumor-suppressing functions); the third was an alteration that has not been previously described: a deleterious hybrid *PMS2* allele produced by recombination with crossover between *PMS2* and *PMS2CL*.

## MATERIAL AND METHODS

### Patient and Reference DNA Samples

This study included gDNA samples from 13 patients with colorectal tumors displaying isolated PMS2 expression loss (Table 1). One (patient TR13) was recruited in the Department of Gastroenterology of the Santa Chiara Hospital in Trento, Italy. The other 12 were enrolled at the Institute of Molecular Cancer Research, University of Zurich, Switzerland. All patients gave written informed consent to molecular genetic testing.

**Table 1:** Patients with Colorectal Tumors

Patient code	Age	Sex	Site	Stage^a^	Grade^a^	MSI	Revised AC and BG
**CH1**	35	M	C	T3N1M0	3	P	BG1 and BG4
**CH2**	29	M	A	T3N0M0	2	Na	BG1
**CH3**	40	F	A	T3N0M0	2	Na	BG1
**CH4**	48	M	A	T2N0M0	3	P	BG1 and BG5
**CH5**	53	M	SF	T3N0M0	2	P	AC
**CH6**	77	M	A	T1N0M0	2	P	Na
**CH7**	73	M	A	T3N0M0	2	P	Na
**CH8**	38	F	C	T3N0M0	2	P	BG1
**CH9**	80	M	A	T3N1M0	3	Na	Na
**CH10**	29	F	S	T2N0M0	2	P	BG1
**CH11**	51	F	SF	T3N0M0	2	P	AC
**CH12**	44	M	R	Advanced adenoma	Severe dysplasia	AB	BG1 and BG5
**TR13**	56	M	C/R	T3N0M0/T1N0M0	G3/G2	P	BG2 and BG5

Note: CH6 = 52557 and CH7 = 61263 in Truninger et al. (2005).

MSI, microsatellite instability at BAT26 and dinucleotide repeats on 7p22; P, present; AB, absent; m, male; f, female; C, cecum; A, ascending; SF, splenic flexure; S, sigmoid colon; R, rectum; AC, revised Amsterdam Criteria; BG, revised Bethesda Guidelines; Na, not available.

a TNM and tumor grade classification according to Sobin and Wittekind (2002).

In the 10 cases in which family history was available, the pedigree fulfilled the revised Amsterdam II criteria (Vasen et al., 1999) (*n* = 2) or one or more of the criteria contained in the revised Bethesda Guidelines (Umar et al., 2004) (*n* = 8). In 11 of the 13 patients, MLPA was the first-line method of mutation analysis. The other two (CH6 and CH7) had undergone gDNA sequencing in a previous study (Truninger et al., 2005), which was negative for point mutations in *PMS2*. DNA remaining after the completion of this study was used now for MLPA analysis.

To identify appropriate reference DNA samples that could be used by all laboratories for *PMS2* MLPA analysis, we obtained 24 DNA samples from Coriell Cell Repositories (Camden, NJ) and analyzed them by MLPA. Results were compared with those for DNA samples from five controls whose genotypes had been determined in a previous study (Ganster et al., 2010). Candidate samples found to contain two *PMS2-*specific copies and two *PMS2CL*-specific copies of each sequence bound by the probes for exons 11-15 were selected as reference samples for this study.

We also used 150 DNA samples from individuals with different ethnic backgrounds (Ganster et al., 2010) as negative controls in the allele-specific PCR assay described below.

### MLPA

The P008-B1 *PMS2* MLPA kit (MRC-Holland) was used according to the manufacturer's instructions to detect single and multiple exon deletions (as well as possible duplications) in the *PMS2* gene. The redesigned assay includes 24 *PMS2*-specific probes (exons 1-15), five *PMS2CL*-specific probes (exons 11-15), and five nonspecific probes (exons 12-15) that hybridize to both *PMS2* and *PMS2CL*. Before MLPA analysis, all gDNA samples were purified with the QIAamp DNA Micro kit (Qiagen, Hilden, Germany), and a total of 50-100 ng of gDNA was used per MLPA reaction. Each MLPA run included five appropriate reference DNA samples (selected as described above; Table 2). MLPA results were analyzed with the Sequence Pilot algorithm, version 3.3 (JSI Medical Systems, Kippenheim, Germany). A relative probe signal of 1 (= 100%) in a sample DNA reflects to two copies of target sequence of *PMS2*- and *PMS2CL*-specific probes and four copies of target sequence of universal probes which bind to both *PMS2* and *PMS2CL* sequences. Hence, relative probe signals of 0.5 (50%) and 1.5 (150%) of *PMS2-* and *PMS2CL*-specific probes indicate one and three DNA copies, respectively, of the respective target sequences. A reduction to 0.75 (75%) indicates a copy number change from four to three copies of target sequence of universal probes.

**Table 2:** Coriell Cell Repository DNA Samples Identified as Valid References for Use with the P008-B1 *PMS2* MLPA Kit

DNA number^a^	Catalog ID	Description
NA07348	GM07348	CEPH/UTAH pedigree 1345
NA10842	GM10842	CEPH/UTAH pedigree 1423
NA07019	GM07019	CEPH/UTAH pedigree 1340
NA12853	GM12853	CEPH/UTAH pedigree 1400
NA17002	GM17002	Human variation panel – Northern European
NA17005	GM17005	Human variation panel – Northern European
NA17008	GM17008	Human variation panel – Northern European
NA17009	GM17009	Human variation panel – Northern European

a Each sample listed was MLPA-verified to contain two copies of *PMS2*-specific and two copies of *PMS2CL*-specific sequences. MLPA profiles of all 24 Coriell DNAs investigated in this study are available on request.

### cDNA Sequencing of *PMS2* and *PMS2CL* Transcripts

This approach was used to confirm MLPA findings of a *PMS2* exon 8 deletion in patient CH11 and those for patient TR13 showing a deleterious hybrid *PMS2* allele containing a *PMS2CL*-derived sequence in exons 11-15. In the former case, we extracted RNA from the patient's freshly collected blood lymphocytes and reverse-transcribed it using the High Capacity RNA-to-cDNA kit (Applied Biosystems, Foster City, CA). cDNA containing *PMS2* exons 6–9 was PCR-amplified with primers PMS2/11 (forward) and PMS2/4 (reverse) (Supporting Information Table S1). These primers were also used to sequence the resulting RT-PCR product. In case TR13, we extracted RNA from a short-term culture of the patient's lymphocytes, which had been treated with puromycin before harvest (Etzler et al., 2008) to prevent nonsense-mediated decay (Andreutti-Zaugg et al., 1997). The entire coding sequence of *PMS2* was amplified in two partially overlapping RT-PCR products, as previously described (Etzler et al., 2008). The amplicons were sequenced with internal primers (Supporting Information Table S1), directly and after cloning into the pCR4-TOPO vector (Invitrogen, Carlsbad, CA). In the latter case, 10 clones were sequenced after amplification of the plasmid inserts by colony PCR performed under standard conditions with Taq DNA polymerase (New England Biolabs GmbH, Frankfurt am Main, Germany) and M13 forward and reverse primers. *PMS2CL* transcripts from the same patient were RT-PCR-amplified and subsequently sequenced as previously described (Ganster et al., 2010).

All sequence reactions were performed with Big Dye Terminator chemistry V1.1 (Applied Biosystems) on the Applied Biosystems 3130 Genetic Analyzer. Before sequencing, PCR products were treated with ExoSAP-IT (GE Healthcare, Vienna, Austria), and sequences were analyzed with the Sequence Pilot algorithm Version 3.3 (JSI Medical Systems, Kippenheim, Germany).

### Determination of the Extent of the Exon 8 Deletion at the gDNA Level

The exon 8-deleted allele from patient CH11 was amplified from gDNA with primers PMS2_7f (forward) and PMS2/4 (reverse), which are located in *PMS2* intron 6 and exon 9, respectively (Supporting Information Table S1). Phusion High-Fidelity DNA polymerase (Finnzymes, Espoo, Finland) was used in a reaction containing 3% DMSO under the PCR conditions recommended by the manufacture. The resulting PCR product (i.e., a single band presumably derived from the deletion-containing allele) was sequenced with forward primer PMS2/3 (Supporting Information Table S1) located within exon 7.

### Genomic Array Analysis of the Deletion Encompassing the Entire *PMS2* Gene and Flanking Sequences

The Affymetrix Genome-Wide Human SNP 6.0 array platform (Affymetrix, Santa Clara, CA) was used according to the manufacturer's instructions for genomic copy number profiling. Probe allocation was based on the March 2006 human reference sequence (NCBI Build 36.1). Raw probe data files were processed within the R statistical software framework (http://www.R-project. org) with additional packages from the aroma. affymetrix project (http://aroma-project.org). Copy numbers were estimated with the CRMAv2 method (Bengtsson et al., 2009), which includes allelic cross-talk calibration, normalization for probe sequence effects, and normalization for PCR fragment-length effects. Copy numbers were segmented using the Circular Binary Segmentation method (Venkatraman and Olshen, 2007). Normalized probe and segmentation data were visualized with custom software developed for the Progenetix project (Baudis and Cleary, 2001).

### Breakpoint Analysis of the Deleterious Hybrid *PMS2* Allele and Development of an Assay for its Detection

The sequence exchange breakpoint responsible for the deleterious hybrid *PMS2* allele in case TR13 was expected to lie in intron 10. Therefore, we specifically amplified a 2,314-bp PCR fragment from gDNA containing the deleterious hybrid allele using a *PMS2*-specific primer located in exon 10 (PMS2B_F) and a *PMS2CL*-specific primer in exon 11 (PMS2CLc.1238CC_R). The fragment was then sequenced with seven internal sequencing primers (Supporting Information Table S1).

We developed a simple PCR-based assay for detecting the deleterious hybrid *PMS2* allele. Primers flanking the identified breakpoint were designed to specifically amplify sequences from the hybrid allele. These primers, that is, a *PMS2*-specific forward primer (PMS2inIVS10_gen_1f) and a *PMS2CL*-specific reverse primer (PMS2inIVS10_psgen_1r), generate a 239-bp hybrid allele-specific PCR product (Supporting Information Table S1). They were used in a duplex PCR reaction with primers that generate a 696-bp control PCR product (PMS2in14_1F and PMS2B_Rnew) (Supporting Information Table S1).

### NCBI Reference Sequences

NCBI reference sequences (RefSeqs) NG_008466.1 and NM_000535.5 were used for the human *PMS2* gene and human *PMS2* mRNA, respectively. RefSeq NC_000007.13 was used for the human *PMS2CL* pseudogene. The sequence variants that discriminate between the *PMS2* and *PMS2CL* paralogs are those published by Hayward et al. (2007); they are given as variants with respect to the *PMS2* RefSeq. All variants are described in accordance with the Human Genome Variation Society (http://www.hgvs.org/mutnomen) guidelines, with the A of the ATG start codon as position c.1.

## Results

### Publicly Available Reference DNA Samples for *PMS2* MLPA Analysis

Because of the high prevalence of nondeleterious hybrid *PMS2* and *PMS2CL* alleles, the distribution of *PMS2* and *PMS2CL*-specific sequences downstream of exon 12 displays high interindividual variability (Hayward et al., 2007;Ganster et al., 2010; van der Klift et al., 2010). Randomly chosen reference samples of DNA are thus likely to differ widely in terms of the distribution of gene- and pseudogene-derived sequences in this region. This is reflected by high standard deviations for the reference DNA signals generated with all the paralog-discriminating probes located downstream of exon 12 (Supporting Information Fig. S1). It is important to note that an unequal distribution of gene-derived and pseudogene-derived sequences in the reference DNA set will reduce the accuracy of copy number assessments at these loci in patient DNA samples. For this reason, reference DNAs must harbor two *PMS2*-specific copies and two *PMS2CL*-specific copies of each sequence bound by paralog-discriminating probes for exons 11-15.

**Figure 1:**
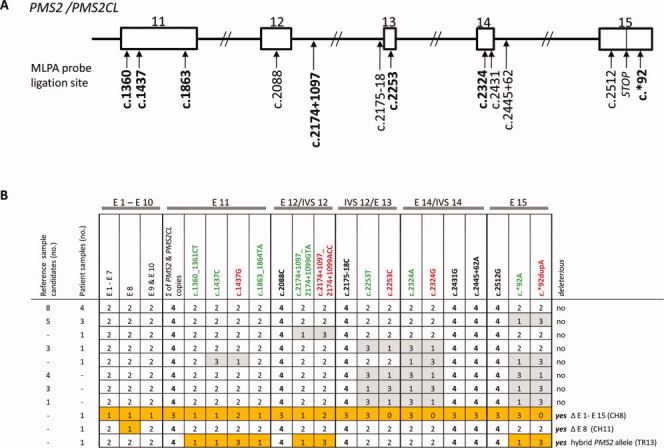
*PMS2* MLPA results for reference DNA candidates and DNA samples from patients with colorectal tumors displaying isolated PMS2 expression loss. (A) Schematic showing ligation sites for the probes in the redesigned (P008-B1) *PMS2* MLPA kit. Paralog-discriminating ligation sites are shown in boldface. Rectangles: exons 11–15 of *PMS2* (and *PMS2CL*); lines: introns. (B) MLPA results (given as copy numbers of the DNA sequences bound by the MLPA probes) for 24 reference DNA sample candidates (obtained from Coriell Cell Repositories) and 13 patient DNA samples. Results are grouped for exons 1–7 (E1-E7) and 9 and 10 (E9 and E10) and reported individually for exons 8 (E8) and exons 11 (E11) to 15 (E15), the latter being shared by the paralogs. The paralog-discriminating sequence variants at the ligation sites of the probes in exons 11–15 are color-coded: green, *PMS2*-specific probes; red, *PMS2CL*-specific probes; black, nonspecific probes. Numbers in cells (0–4): copies of exon sequences in sample. Paired gray cells: uneven distribution of *PMS2*- and *PMS2CL-*specific signals in samples with a total of four copies, which reflects the presence of nondeleterious hybrid alleles (*PMS2* or *PMS2CL*). Orange cells: aberrant MLPA results in patient CH8 (reflecting deletion of all *PMS2* exons); patient CH11 (exon 8 deletion); and patient TR13 (who had a deleterious *PMS2* hybrid allele; see text for details). [Color figure can be viewed in the online issue, which is available at wileyonlinelibrary.com.]

To obtain a set of reference samples that could be used by all laboratories performing *PMS2* MLPA, we evaluated 24 DNA samples from EBV-immortalized lymphocytes, which can be obtained from Coriell Cell Repositories. MLPA findings for these candidates were compared with those for five previously characterized DNA samples (Ganster et al., 2010) that met the criteria listed above. Sixteen (67%) of the candidates showed MLPA signals reflecting an unequal distribution of gene-derived and pseudogene-derived sequences (Fig. 1). The other eight (33%) met the prerequisites for suitable reference samples (Table 2). Each subsequent MLPA experiment was performed with a control set consisting of five of these samples (randomly chosen for each experiment).

### Alterations Identified by *PMS2* MLPA Analysis of gDNA from Patients with PMS2-Deficient Colorectal Tumors

As shown in Figure 1B, aberrant MLPA signals indicating a deleterious *PMS2* alteration were detected in three (23%) of the 13 patients with PMS2-deficient colorectal tumors (Table 1). In Patient CH8, the assay revealed loss of one *PMS2*-specific DNA copy of exons 1-12 and the presence of two *PMS2CL*-specific DNA copies in exons 11 and 12 (Fig. 1B; Supporting Information Fig. S2). We found reduced probe signals indicative for three (instead of four) copies of the sequences bound by the nonspecific probes for exons 12, 13, 14, and 15. Paralog-specific probe signals for the latter three exons disclosed that the three copies were all *PMS2*-derived (Fig. 1B, Supporting Information Fig. S2). This MLPA pattern is most likely the result of a deletion encompassing the entire *PMS2* gene and the concomitant presence of two hybrid *PMS2CL* alleles containing *PMS2*- specific sequences in exons 13-15. The latter alleles, together with the remaining nonhybrid, wild-type *PMS2* allele, would account for the three gene-specific signals in exons 13-15.

Genomic array analysis (Supporting Information Fig. S3) confirmed the presence of a large (∼125 kb) deletion containing the entire *PMS2* gene, three other genes (*ANKRD61*, *AIMP2* [*JTV1*], and *EIF2AK1*), and a portion of the coding region of *RSPH10B*. The distal breakpoint of the deletion was located downstream to *PMS2*, in the center of the telomeric duplicon, between probes CN_1240840 and CN_1240842 [at nucleotides 5,970,140 and 5,970,432 (NCBI build 36.1/hg18), corresponding to between 6,003,614 and 6,003,906 in GRCh37/hg19]. The proximal breakpoint lay within the interval of nucleotides 6,095,502–6,097,302; probes CN_1240873 and CN_1240874 (corresponding to 6,121,772–6,137,980 in hg19, respectively) which is located within the 0.7-Mb sequence separating the telomeric and centromeric duplicon. Interestingly, this breakpoint falls within the range reported for a deletional CNV that has been observed in a number of studies (e.g., Kidd et al., 2008).

In patient CH11, MLPA showed loss of one DNA copy at the site hybridized by the exon 8 probe (Fig. 1B). Sequencing of exon 8 from gDNA excluded sequence alterations at this site, whereas sequencing of an RT-PCR product comprising *PMS2* exons 6–9 revealed heterozygous loss of exon 8 in the patient's transcripts (data not shown). Collectively, these results confirmed the presence of a genomic deletion involving *PMS2* exon 8.

To characterize better this deletion, we amplified patient gDNA using a forward primer (PMS2_7f) located in intron 6 and a reverse primer (PMS2/4) in exon 9. The amplicon produced a single band in agarose gel electrophoresis and was ∼3,400 shorter than the expected product of wild-type allele amplification (∼2,200 vs. 5,597 bp), which indicates that only the deletion-carrying allele is amplified under the PCR conditions we used. Sequencing of this amplicon with an internal primer showed that the breakpoints of this 3,351-bp deletion (i.e., c.803+384_904-1533del) were located within two identically oriented *Alu*Sb elements in introns 7 and 8.

**Figure 2:**
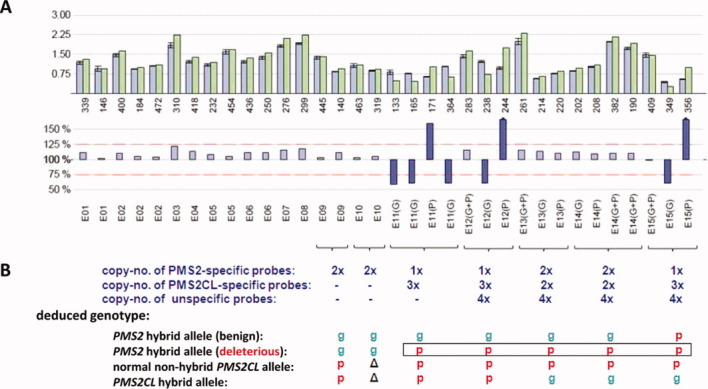
MLPA results showing the deleterious hybrid *PMS2* allele in patient TR13. (A) Bars in upper and lower histograms represent MLPA probes. Probe labels appear under the lower histogram. Probes for exons (E) 11–15 are designated gene-specific (G), pseudogene-specific (P), or nonspecific (G+P). Upper histogram: Lavender bars indicate mean probe signals with standard deviations for five of the selected reference DNAs reported in Table 2; green bars: probe signals for patient DNA. Numbers below the green bars indicate the amplicon size (in nts) of the corresponding MLPA probe. Lower histogram: the bar for each probe represents the probe signal for patient DNA as a percentage of the mean signal for the reference DNAs. Lavender bars represent percentages ranging from 75 to 125% (red dotted lines); larger discrepancies between patient and reference samples are represented by violet bars. (B) Number of DNA copies at sites hybridized by probes for exons 11–15 and deduced patient genotypes. Green “g”: *PMS2*-specific sequence; red “p”: *PMS2CL*-specific sequence; Δ: absent in *PMS2CL* (exon 10) (see text for explanation of these results). [Color figure can be viewed in the online issue, which is available at wileyonlinelibrary.com.]

MLPA results for the third patient (TR13) revealed one gene-specific and three pseudogene-specific DNA copies at all probe sites in exons 11, 12, and 15 (Fig. 1B). In exons 13 and 14, the patient harbored two *PMS2*- and *PMS2CL*-specific copies (Figs. 1B and 2A). These findings are indicative of a hybrid *PMS2* allele containing a *PMS2CL*-derived sequence, which starts at the beginning of exon 11 (position c.1360_1361, where the first *PMS2*-specific MLPA probe in exon 11 is located; see Fig. 1A) and extends at least 1,099 bp downstream from exon 12 (specifically, to position c.2174+1097_2174+1099, where the gene-specific and pseudogene-specific intron 12 probes are located; see Fig. 1A). Compared with its *PMS2* paralog, the *PMS2CL* exon 11 sequence contains two frame-shift variations (c.1730dupA and c.1863_1864delTA). As each of these variations produces a premature stop codon when introduced into *PMS2*, the hybrid allele can be classified as deleterious.

We suspected that the *PMS2CL*-derived sequence in this hybrid allele extended even farther downstream, into or past exon 15, and that the equal distribution of gene- and pseudogene-specific sequences (2:2) in exons 13 and 14 might reflect the presence of a hybrid *PMS2CL* allele containing *PMS2*-specific sequences in these exons. To confirm this hypothesis, we sequenced *PMS2* transcripts from the patient that had been amplified as described by Etzler et al. (2008) using a forward primer located in exon 10 and a reverse primer downstream of the termination codon. Direct sequencing of the RT-PCR products (Supporting Information Fig. S4) showed heterozygosity for all known *PMS2CL*-specific sequence variants in exons 11–14 and homozygosity for the *PMS2CL*-specific variant c.*92dupA in exon 15. When individual RT-PCR products were sequenced after cloning, the results confirmed the presence of the deduced deleterious *PMS2* hybrid allele (with sequences in exons 11–15 that were all *PMS2CL*-derived). They also showed that the other *PMS2* allele was a nondeleterious hybrid containing the pseudogene-derived variant c.*92dupA. Moreover, cDNA sequencing of *PMS2CL* transcripts confirmed the presence of a nonhybrid allele, together with the expected hybrid *PMS2CL* allele containing *PMS2*-specific sequences in exons 13–15 (data not shown). This complex genotype (Fig. 2B) explains the gene:pseudogene copy number ratios observed in exons 11, 12, and 15 (1:3) and in exons 13 and 14 (2:2).

### Characterization of the Breakpoint of Sequence Exchange Between *PMS2* and *PMS2CL* Leading to the Deleterious *PMS2* Hybrid Allele

**Figure 3:**
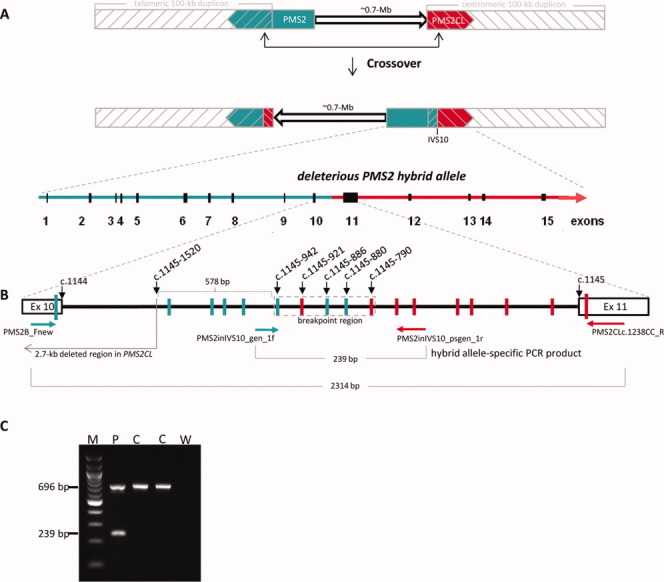
Breakpoint analysis and allele-specific PCR detection of the deleterious hybrid *PMS2* allele in patient TR13. (A) Schematic showing sequence exchange between *PMS2* and *PMS2CL* due to intrachromosomal recombination with crossover. Upper figure: Normal locations on 7p22 of gene (green); pseudogene (red); and 100-kb duplicons (gray hatching). Arrowheads indicate direction of transcription. The 0.7-Mb interduplicon sequence is shown as an arrow. Middle figure: intrachromosomal recombination with crossover produces a hybrid *PMS2CL* allele (in the telomeric duplicon) containing a *PMS2*-derived sequence and a hybrid *PMS2* allele (partially embedded in the centromeric duplicon) that contains a *PMS2CL*-derived sequence 3′ of the recombination breakpoint (intron 10; IVS10). The interduplicon sequence has also been inverted. Lower figure: zoom showing the hybrid *PMS2* allele with *PMS2*-derived (green) and *PMS2CL*-derived (red) sequences. (B) Zoom (not to scale) showing the breakpoint region of the deleterious hybrid *PMS2* allele. The heavy black line between exons 10 and 11 (rectangles) represents intron 10. Vertical bars represent *PMS2*-derived (green) and *PMS2CL*-derived (red) sequence variants. Horizontal arrows represent allele-specific primers spanning the breakpoint (gene-specific in green, pseudogene-specific in red). The resulting allele-specific PCR products (2,314 and 239 bp long) are indicated (light gray brackets), and the breakpoint region is enclosed in a dotted rectangle (light gray). (C) Results of the duplex PCR assay for the deleterious hybrid *PMS2* allele in patient TR13 (P) and two of the 150 normal controls (C) tested. The 696-bp control PCR product was present in the patient and all 150 controls; the 239-bp PCR product specific for the deleterious hybrid *PMS2* was present only in the patient sample. M: 100 bp DNA ladder; W: negative (water) control. [Color figure can be viewed in the online issue, which is available at wileyonlinelibrary.com.]

Recombination with crossover between the paralogous sequences is the most likely mechanism underlying the formation of the deleterious hybrid *PMS2* allele (Fig. 3A). Because the recombination breakpoint was expected to lie 5′ of exon 11, in intron 10, we amplified a 2314-bp fragment containing this intron 10 specifically from the deleterious hybrid allele. For this purpose, we used a gene-specific forward primer located in exon 10 and a pseudogene-specific reverse primer in exon 11. As shown in Figure 3B, sequencing of the amplicon revealed *PMS2*-specific single-nucleotide variants at all paralog-discriminating sites 5′ of nucleotide c.1145-942 (inclusive) and *PMS2CL*-specific single-nucleotide variants 3′ of nucleotide c.1145-790 (inclusive). These two nucleotides delimit the 153-bp breakpoint region, which includes three additional paralog-discriminating, single-nucleotide variants: one derived from *PMS2CL* (the adenine at position c.1145-921) and two from *PMS2* (the guanine and inserted cytosine at positions c.1145-886 and c.1145-880_1145-879, respectively; Fig. 3B). This pattern of gene- and pseudogene-specific variants within the breakpoint region reflects the type of double Holliday junction resolution that leads to strand crossover in the recombination-initiated, double-strand break repair process (as well-illustrated in Fig. 1 of Chen et al., 2007), thereby confirming that recombination with crossover is the mechanism that generated this deleterious hybrid *PMS2* allele.

Because *PMS2* and *PMS2CL* are oppositely oriented, interparalog crossover is more likely to occur intrachromosomally. This process would generate two reciprocal hybrids, each containing paralog-derived sequences (Fig. 3A). However, in the presence of a reciprocal hybrid *PMS2CL* allele, the deleterious hybrid *PMS2* allele found in patient TR13 would have escaped detection by MLPA because of the equal numbers of gene-specific and pseudogene-specific sequences (2:2) in exons 11 and 12. *PMS2*-specific cDNA sequencing (Etzler et al., 2008; van der Klift et al., 2010) as well as approaches based on *PMS2*-specific long-range PCR (Vaughn et al., 2010) would detect the deleterious hybrid allele also in this conformation. However, high quality RNA and gDNA is needed for these assays. As such material is frequently not available of retrospective patient's cohorts, we developed a simple PCR assay that identifies this deleterious hybrid allele even when it is accompanied by its reciprocal hybrid *PMS2CL* allele. This assay can be used as screening tool in large retrospective cohorts. It is based on the use of primers that specifically amplify a 239-bp breakpoint-including fragment from the deleterious *PMS2* hybrid allele (Fig. 3B). This reaction is duplexed with a control PCR that generates a 696-bp fragment using nonspecific primers located in exon 15 (Ganster et al., 2010). After verifying its specificity in the analysis of DNA samples from 150 control individuals (Ganster et al., 2010), all of which were negative for the 239-bp long PCR product (Fig. 3C), we used the new assay to retest the 10 patients in our cohort whose suspected *PMS2* mutation had not been verified. Negative findings in all 10 cases exclude the possibility that a reciprocal *PMS2CL* hybrid allele is masking the presence in these individuals of the deleterious hybrid *PMS2* allele found in patient TR13.

## Discussion

The high prevalence of clinically irrelevant *PMS2* and *PMS2CL* hybrid alleles (found in ∼70% in the Caucasian population; van der Klift et al., 2010) has seriously hindered MLPA-based detection of copy number changes involving *PMS2* exons 13–15. Our findings confirm that *PMS2* MLPA analysis can be substantially improved by the use of MRC Holland's recently redesigned *PMS2* MLPA kit (P008-B1), together with selected reference DNA samples known to carry two *PMS2-*derived and two *PMS2CL*-derived sequences in this region (Vaughn et al., 2011). The eight reference samples we verified for use in this study can be adopted as controls for this assay in any diagnostic laboratory. They are publicly available (from Coriell Cell Repositories), and because they are derived from EBV-immortalized lymphocyte cell lines, their availability is long-term. Routine use of these reference DNAs can facilitate standardization of MLPA analysis.

With this improved MLPA approach, we analyzed germline DNA from 13 patients with colorectal tumors characterized by isolated PMS2 expression loss. The assay identified deleterious germline alterations involving the *PMS2* locus in 3 (27%) of the 11 cases in which MLPA was the first-line method of mutation analysis (23% of the entire cohort). These figures are consistent with previous MLPA findings in colon cancer series similar to our own. Deleterious *PMS2* copy number alterations were found in 11 (17%) of 65 patients tested in a US laboratory (Vaughn et al., 2010, 2011) whereas a Dutch group reports that up to 33% (15/45) of the analyzed patients carry germline *PMS2* mutations that should be detectable with the P008-B1 *PMS2* MLPA kit (van der Klift et al., 2010; van der Klift and Tops, personal communications).

To gain new insight into the mechanisms that generate *PMS2* mutations, we characterized the alterations found in our cohort in greater detail. The 3,351-bp deletion that included *PMS2* exon 8 (Patient CH11) was probably caused by recombination of two identically oriented *Alu*Sb elements in introns 7 and 8. Together with the two other previously described *Alu* recombination-mediated deletions, our finding strengthens the view that the frequency of deletions involving *PMS2* is related in part to the high density of *Alu* elements within the genomic sequence of this gene (van der Klift et al., 2005).

The deletion found in patient CH8 was much larger (∼125 kb). It included the entire *PMS2* gene and at least three other genes as well. Two of these genes (*EIF2AK1* and *AIMP2*) have tumor-suppressing functions. EIF2AK1 suppresses protein synthesis during various conditions of stress by blocking the initiation of translation. This inhibition has been shown to annul the proliferative capacity of differentiating erythroid cells (Crosby et al., 2000), and similar effects might occur in colorectal tissues, where *EIF2AK1* is highly expressed (our data, not shown).

As for the second gene, *AIMP2*, its promoter region lies head-to-head with and partially overlaps that of *PMS2* (NCBI RefSeq NG_008466.1; Nicolaides et al., 1995). The expression of this gene in colorectal tissues is even more abundant than that of *PMS2* (our data, not shown): it encodes a component of the amino-acyl-tRNA synthetase complex (Kim et al., 2002), which exerts both antiproliferative and proapoptotic activities in diverse cell types. On TGF-β stimulation, AIMP2 mediates ubiquitination and degradation of FUBP1, a transcriptional activator of the proto-oncogene *MYC* (Kim et al., 2003). Furthermore, DNA damage due to genotoxic stress is followed by phosphorylation of AIMP2, which then promotes apoptosis by protecting TP53 from MDM2-mediated ubiquitination and degradation (Han et al., 2008). AIMP2 also promotes TNF-α-dependent apoptosis via ubiquitination-mediated degradation of TRAF2 (Choi et al., 2009a). Most importantly, reduced AIMP2 expression level in *Aimp2* heterozygous mice is associated with increased susceptibility to cancer in the colon and other tissues as well (Choi et al., 2009b). *AIMP2* is probably involved in all *PMS2* deletions affecting exon 1 and sequences 3′ to this exon. Thus far, *PMS2* deletions of this type have been reported in eight further families (Hendriks et al., 2006; Overbeek et al., 2007; Senter et al., 2008; Vaughn et al., 2010; Herkert et al., 2011). It may be interesting to investigate in future studies whether inactivation of the *AIMP2* gene contributes as a modifier to the expression of Lynch syndrome.

The deleterious alteration identified in patient TR13 was a hybrid *PMS2* allele generated by recombination with crossover, one possible resolution of a double Holliday junction. The recombination breakpoint was located in *PMS2* intron 10, just 578 bp 3′ of a 2.7-kb sequence that is present in *PMS2* and deleted in *PMS2CL.* This major dissimilarity between gene and pseudogene (Fig. 3B) impedes alignment of the paralogous sequences at the inner ends of the duplicons; consequently, the rate of interparalog recombination should be very low in this region (Fig. 3A) (Hayward et al., 2007). Indeed, of all the breakpoints reported thus far to cause sequence exchange between the human *PMS2* and *PMS2CL* genes, the one that generated the deleterious hybrid *PMS2* allele found in patient TR13 is the most proximal (i.e., closest to the inner ends of the duplicons)*.* The next closest – located approximately 1,500 bp downstream from the breakpoint we describe – also generated a deleterious hybrid *PMS2* allele associated with Lynch syndrome (Auclair et al., 2007). It contained two adjacent *PMS2CL*-specific sequence variants (c.1730dupA and c.1732C>T) in exon 11, which were probably inserted by the nonreciprocal mechanism of gene conversion, another possible result of the resolution of a double Holliday-junction (Auclair et al., 2007). This hybrid allele and the one found in patient TR13 are the only deleterious *PMS2* alterations reported thus far that have been attributed to sequence exchange between *PMS2* and *PMS2CL*. This confirms previous observations that interparalog recombination events located 5′ to intron 12 are much less common than those occurring downstream of exon 12 (Hayward et al., 2007; Ganster et al., 2010; van der Klift et al., 2010).

We recently characterized a nondeleterious hybrid *PMS2* allele produced by recombination with crossover with a breakpoint in intron 12 (Ganster et al., 2010). As discussed above and shown for this hybrid allele, intrachromosomal recombination resulting in crossover of paralog sequences is expected to produce reciprocal hybrid *PMS2* and *PMS2CL* alleles (Fig. 3A).

**Figure 4:**
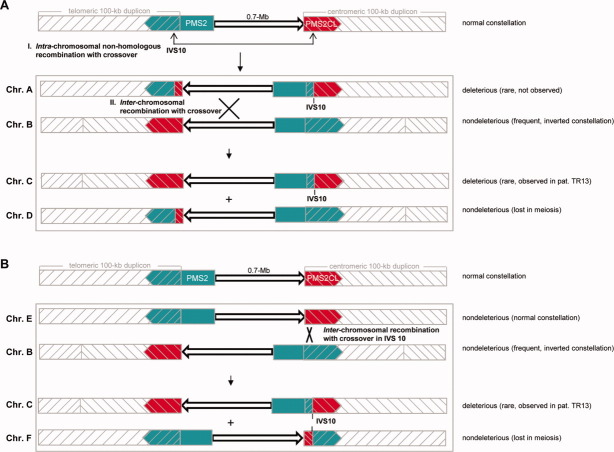
Possible mechanisms leading to the configuration found in patient TR13: a deleterious hybrid *PMS2* allele with no reciprocal hybrid *PMS2CL* allele. The normal configuration depicted at the tops of panels A and B is that shown in Figure 3A (see legend of Fig. 3 for meaning of colors and symbols). (**A**) First hypothesis: Event I. Intrachromosomal recombination with crossover generates chromosome A (Chr.A) containing both the deleterious hybrid *PMS2* allele and a reciprocal hybrid *PMS2CL* allele. The recombination breakpoint is located in intron 10 (IVS10). Event II (occurring simultaneously with or after Event I). Interchromosomal recombination with crossover (the breakpoint is in the 0.7-Mb interduplicon sequence) between Chr.A and Chr.B (characterized by a common inverted configuration that is nondeleterious) gives rise to the configuration observed in patient TR13 (Chr.C): the deleterious hybrid *PMS2* allele and a normal *PMS2CL* allele. (B) Second hypothesis: interchromosomal recombination event between Chr.B (as described in panel A) and a chromosome with the normal configuration (Chr.E). With the recombination breakpoint located within intron 10, this sequence exchange generates the deleterious *PMS2* hybrid allele found in patient TR13 (Chr.C). [Color figure can be viewed in the online issue, which is available at wileyonlinelibrary.com.]

But, the expected hybrid *PMS2CL* allele was not found in patient TR13 or any of his relatives. Indeed, in this family, the deleterious hybrid *PMS2* allele and the nonhybrid *PMS2CL* allele segregate together, as shown by MLPA analysis of the proband's mother, one of his maternal cousins, and the latter's son (all mutation carriers) and four unaffected individuals, including the proband's daughter and brother (data not shown). These two alleles are thus located on the same chromosome. This finding is compatible with two possible mechanisms of hybrid allele formation (Fig. 4).

In the first hypothesis (Fig. 4A), an initial intrachromosomal recombination with crossover event generated the deleterious hybrid *PMS2* allele. This allele was later separated from its reciprocal hybrid *PMS2CL* allele by an interchromosomal recombination event with a chromosome carrying a nonhybrid *PMS2* allele and a nonhybrid *PMS2CL* allele separated by a 0.7-Mb sequence whose orientation was inverted with respect to the normal conformation – a situation observed in at least 5% of all Caucasians (Feuk et al., 2005). If this mechanism generated the deleterious *PMS2* hybrid allele, the interchromosomal recombination event must have taken place at least two generations before that of the proband and several generations after the intrachromosomal recombination event (more details below). Alternatively, the two steps might have been completed simultaneously, in a single, complex recombination event occurring at least one generation before that of the proband's mother.

In the second hypothesis (Fig. 4B), there is only one step: an interchromosomal recombination event between two chromosomes with *PMS2* and *PMS2CL* in opposite duplicons and separated by oppositely oriented 0.7-Mb sequences.

These considerations may have also diagnostic implications. If the mechanism illustrated in Figure 4B is responsible for its formation, the deleterious hybrid *PMS2* allele found in patient TR13 might have arisen very recently. The same is true if it was generated as shown in Figure 4A with simultaneous intrachromosomal and interchromosomal recombination events. In either case, the deleterious hybrid allele would be very rare in our proband's population. In contrast, if the intrachromosomal and interchromosomal events depicted in Figure 4A occurred successively rather than simultaneously, they were probably separated by an interval of more than 130 generations (assuming an average recombination rate of 1.1 cM Mb^−1^ for the 0.7-Mb sequence between the duplicons; Kong et al., 2002). And during this interval, the reciprocal hybrid alleles would have spread through a considerable portion of the population, in tandem. This latter mutational configuration, however, would escape detection by MLPA analysis (see “Results” Section), but, it could be identified by *PMS2*-specific cDNA sequencing or by the deleterious hybrid allele-specific multiplex assay we developed (Figs. 3B and 3C).

Future studies using one or both of these assays to test patients with colon cancers characterized by isolated loss of PMS2 expression and with negative results at first-line mutation analysis will shed light on the true frequency of this deleterious hybrid allele and the possibility that it represents a founder mutation in the population inhabiting the eastern Alpine valleys of Italy (where patient TR13 comes from). In turn, identification of this allele in other patients could provide us with additional information on the recombination mechanism that might have generated it.

In conclusion, using the P008-B1 *PMS2* MLPA kit and a set of selected reference DNAs that are universally available, we identified deleterious *PMS2* alterations in 23% of our PMS2-deficient colorectal cancer patients (27% of those undergoing first-line testing). The alterations found included two deletions (confirming the high frequency of this type of alteration) and a deleterious hybrid *PMS2* allele, which is particularly interesting since almost all *PMS2-PMS2CL* hybrids described thus far have been nondeleterious. These findings suggest that MLPA should be considered a possible first-line method for *PMS2* mutation analysis, especially when high-quality RNA is not available for direct cDNA sequencing.
