# Supplementary figures and images for: Improved Multiplex Ligation-Dependent Probe Amplification Analysis Identifies a Deleterious PMS2 Allele Generated by Recombination with Crossover Between PMS2 and PMS2CL
Source: Genes Chromosomes Cancer. 2012 May 14;51(9):819–31. doi: 10.1002/gcc.21966 (PMC3398144; doi:10.1002/gcc.21966)

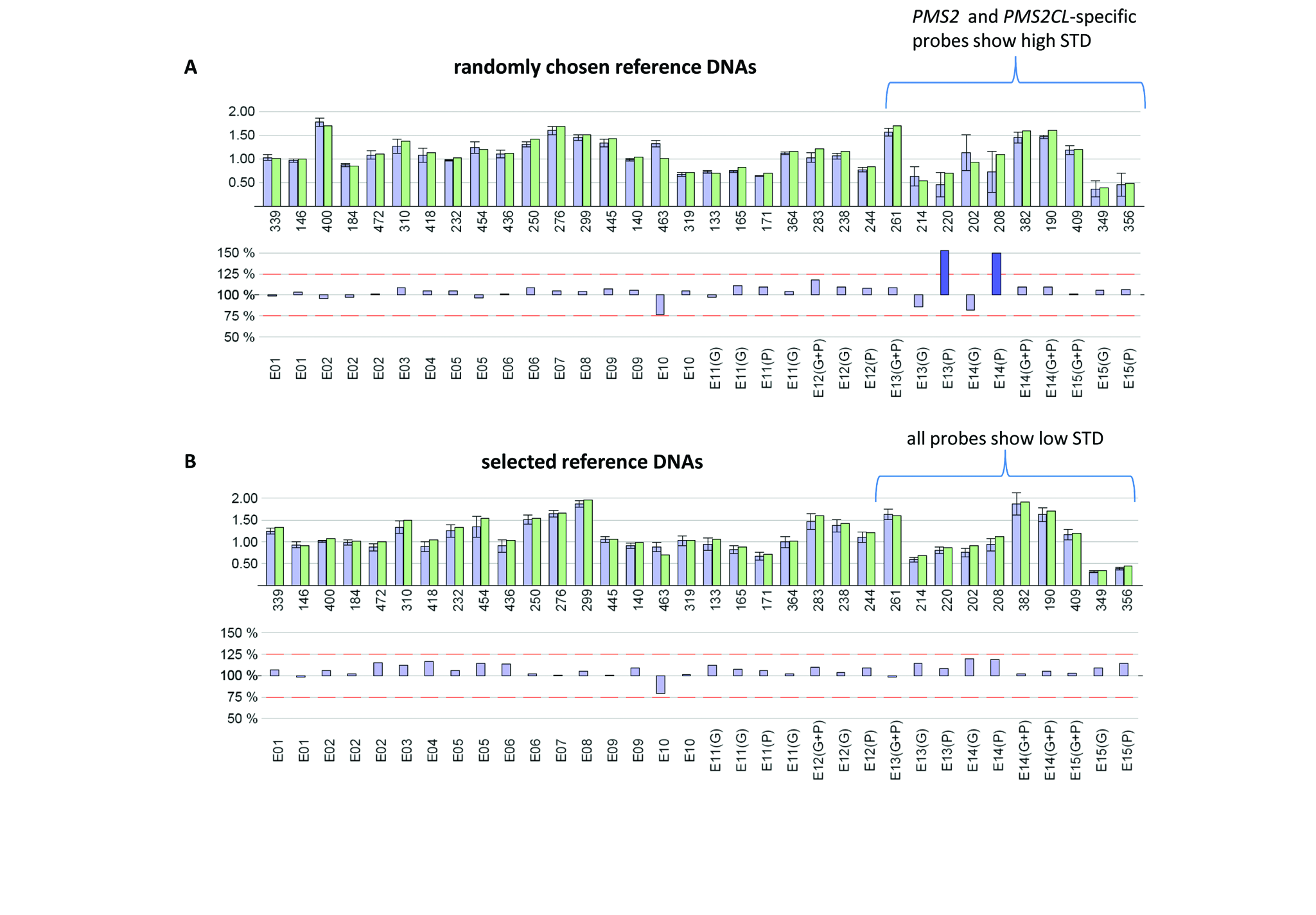

Supplement: Supplementary file 1 [file gcc0051-0819-SD1.tif]

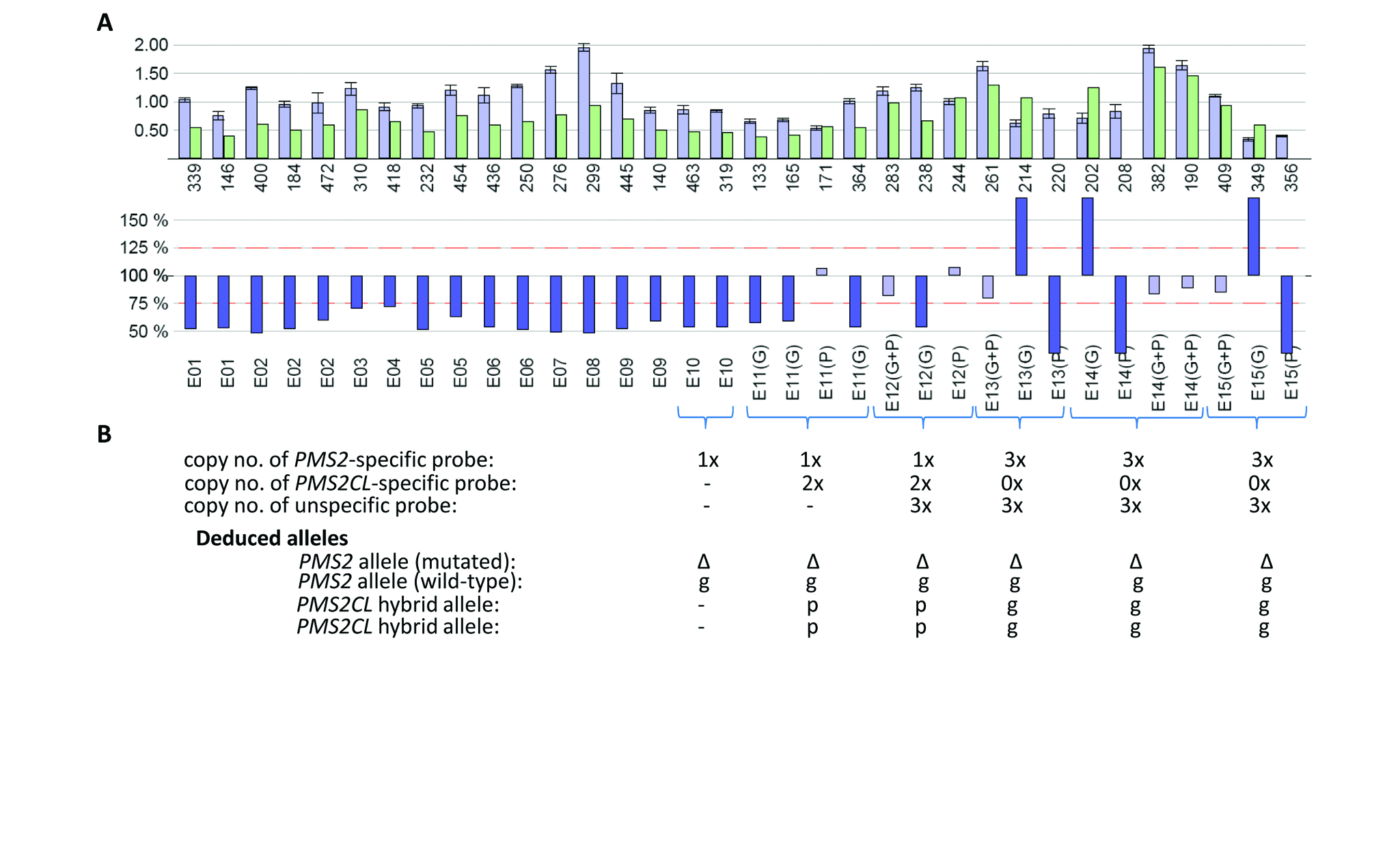

Supplement: Supplementary file 2 [file gcc0051-0819-SD2.tif]

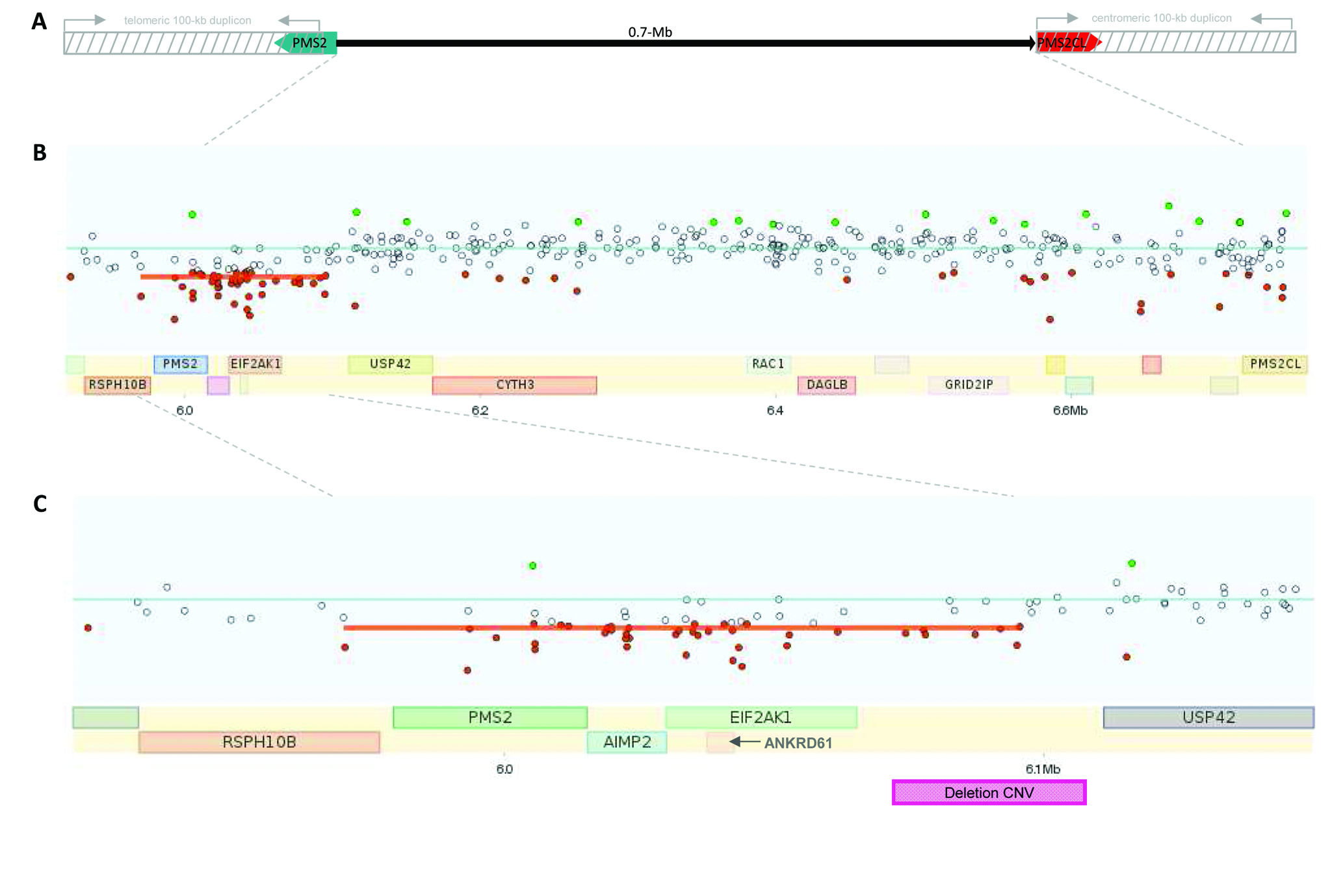

Supplement: Supplementary file 3 [file gcc0051-0819-SD3.tif]

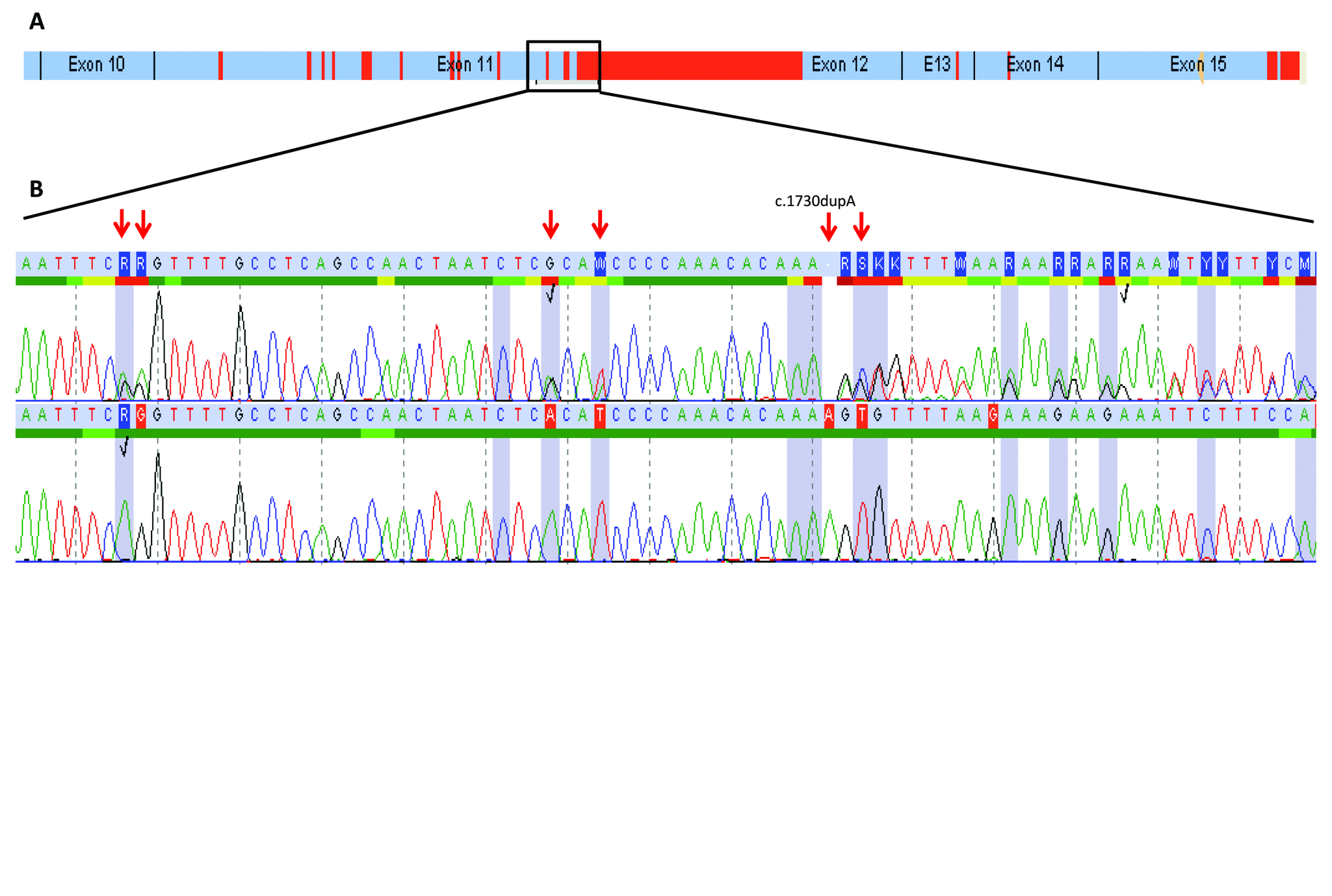

Supplement: Supplementary file 4 [file gcc0051-0819-SD4.tif]
