# Supplementary material for: Improved Multiplex Ligation-Dependent Probe Amplification Analysis Identifies a Deleterious PMS2 Allele Generated by Recombination with Crossover Between PMS2 and PMS2CL
Source: Genes Chromosomes Cancer. 2012 May 14;51(9):819–31. doi: 10.1002/gcc.21966 (PMC3398144; doi:10.1002/gcc.21966)
Supplement: Supplementary file 5 [file gcc0051-0819-SD5.doc]

| **Supplementary Table 1: Primers Used in the Study** | | | | |
| --- | --- | --- | --- | --- |
| **Name [references^a^]** | **Location**^b^ | **Direction**^c^ | **Use**^d^ | **Sequence** |
| **For characterization of the *PMS2* exon 8 deletion:** | | | | |
| PMS2/11 | E5/E6 | Fwd | PCR/Seq | 5‘- ggaatattaagaaggagtatgcc -3‘ |
| PMS2/4 | E9 | Rev | Seq/Seq | 5‘- caacaaatggatactggtgtcg -3‘ |
| PMS2_7f [2] | IVS6 | Fwd | PCR | 5‘- AAAGAAAACAGATTAAGTCC -3‘ |
| PMS2/3 | E7 | Fwd | Seq | 5‘- GACTCCGTGTGTGAAGAGTACGG -3‘ |
| **For characterization of the deleterious hybrid *PMS2* allele:** | | | | |
| PMS2A_F [1] | E1 | Fwd | PCR | 5’- ggatcgggtgttgcatc -3’ |
| PMS2A_R [1] | E11 | Rev | PCR/Seq | 5’- ctttctcctgagagtccacatg -3’ |
| PMS2B_Fnew [1,3] | E10 | Fwd | PCR | 5’- gcagccactgctggatgttgaag -3’ |
| PMS2B_R [1,3] | E15 | Rev | PCR | 5’- ggtttgaaaaggttctcaagatcac -3’ |
| PMS2CLB_F [3] | E9/E11 | Fwd | PCR | 5’- CTTaacatttctgttgattcaggtaac -3’ |
| PMS2/1_2 | E4/E5 | Rev | Seq | 5’- gtagaaatggtgacatcgctc -3’ |
| PMS2/2_2 | E6 | Rev | Seq | 5’- acttacacggatgcctgctg -3’ |
| PMS2/3 | E7 | Fwd | Seq | 5’- gactccgtgtgtgaagagtacgg -3’ |
| PMS2/4 | E9 | Rev | Seq | 5‘- caacaaatggatactggtgtcg -3‘ |
| PMS2/6 [3] | E11 | Fwd | Seq | 5’- tctgacaaaggcgtcctgag -3’ |
| PMS2/7 [3] | E11 | Fwd | Seq | 5’- tctcaggttgatgtagctgtg -3’ |
| PMS2/8 [3] | E12/E13 | Fwd | Seq | 5’- ggctcatagcacctcagactctc -3’ |
| PMS2c.1238CC_R | E11 | Rev | PCR | 5‘- ctggaaatggacacgtccc -3‘ |
| PMS2_1238_1f | IVS10 | Fwd | Seq | 5‘- actgtaaacggatgaagtc -3‘ |
| PMS2_1238_1r | IVS10 | Rev | Seq | 5‘- agatcgcatcattgcactcc -3‘ |
| PMS2_1238_2f | IVS10 | Fwd | Seq | 5‘- aggctggtctcaaactcttg -3‘ |
| PMS2_1238_3f | IVS10 | Fwd | Seq | 5‘- tttgaagttattcagtctgtcc -3‘ |
| PMS2_1238_4f | IVS10 | Fwd | Seq | 5‘- tttgagagggagtctcactc -3‘ |
| PMS2_1238_2r | IVS10 | Rev | Seq | 5‘- caaatgctcacaaagatcagg -3‘ |
| PMS2_1238_5f | IVS10 | Fwd | Seq | 5‘- tgagacagagtgttgctctg -3‘ |
| PMS2inIVS10_gen_1f | IVS10 | Fwd | PCR | 5‘- ctttttgacgagcatagatagg -3‘ |
| PMS2inIVS10_psgen_1r | IVS10 | Rev | PCR | 5‘- accaacatgctgaaacctcg -3‘ |
| PMS2in14_1F [3] | IVS14 | Fwd | PCR | 5‘- gaagtggaatgaataacctgacag -3‘ |
| ^a^ References: [1] Etzler et al., 2008; [2] Hendriks et al., 2006; [3] Ganster et al., 2010  ^b^ IVS = intron, E = exon  ^c^ Fwd = forward, Rev = reverse  ^d^ PCR = polymerase chain reaction, Seq = sequencing | | | | |
